# Bioengineered extracellular vesicles integrating anti-EphA2 recognition and oxaliplatin delivery for pancreatic cancer therapy

**DOI:** 10.1080/10717544.2026.2728215

**Published:** 2026-09-22

**Authors:** Shi-Wei Chao, Wan-Chi Tsai, Yung-Han Chang, Shan-Jou Chen, Chia-Tse Li, Jo-Han Fan, Bo-Cheng Huang, Shih-Ting Hong, Hong-Ren Wang, Yu-Ting Huang, Ming-Yii Huang, Chih-Hung Chuang

**Affiliations:** a Graduate Institute of Medicine, College of Medicine, Kaohsiung Medical University, Kaohsiung, Taiwan; b Drug Development and Value Creation Research Center, Kaohsiung Medical University, Kaohsiung, Taiwan; c Department of Medical Laboratory Science and Biotechnology, Kaohsiung Medical University, Kaohsiung, Taiwan; d Department of Laboratory Medicine, Kaohsiung Medical University Hospital, Kaohsiung, Taiwan; e Department of Radiation Oncology, Kaohsiung Medical University Hospital, Kaohsiung Medical University, Kaohsiung, Taiwan; f Department of Radiation Oncology, School of Medicine, College of Medicine, Kaohsiung Medical University, Kaohsiung, Taiwan; g Center for Cancer Research, Kaohsiung Medical University, Kaohsiung, Taiwan

**Keywords:** Extracellular vesicles, EphA2, oxaliplatin, targeted drug delivery, pancreatic ductal adenocarcinoma

## Abstract

Pancreatic ductal adenocarcinoma (PDAC) remains one of the most lethal solid tumors due to its dense desmoplastic stroma, poor vascular perfusion, and the limited intratumoral delivery of chemotherapeutic agents such as oxaliplatin (OXA). Extracellular vesicles (EVs) offer a biocompatible platform for drug delivery, yet their intrinsic lack of tumor specificity constrains therapeutic efficacy. Ephrin type-A receptor 2 (EphA2), which is highly expressed and efficiently internalized in PDAC, represents an attractive molecular target for guiding EVs into tumor cells. In this study, we engineered HEK293T-derived EVs to display membrane-anchored anti-EphA2 Fab fragments and encapsulate OXA (EVs-EphA2/OXA) as a targeted delivery system for PDAC therapy. Stable producer cells expressed the engineered Fab on the plasma membrane and released vesicles that retained canonical EV markers and robust antigen-binding capability. EVs-EphA2 demonstrated selective uptake into EphA2-positive AsPC-1 and BxPC-3 cells and induced significantly greater cytotoxicity than free OXA or untargeted EVs/OXA in vitro. In xenograft models, EVs-EphA2/OXA achieved the most pronounced tumor suppression, accompanied by increased γH2AX-associated DNA damage, enhanced TUNEL-positive apoptosis, and preferential tumor accumulation in biodistribution imaging. These findings demonstrate that EphA2-targeted EVs can substantially improve intratumoral delivery of OXA and amplify antitumor efficacy, supporting EVs-EphA2/OXA as a promising platform for receptor-guided chemotherapy in PDAC.

## 1. Introduction

Few cancers are as relentlessly lethal as pancreatic ductal adenocarcinoma (PDAC), a malignancy that remains asymptomatic until late stages and responds poorly to current therapies (Bray et al. 2024). With a five-year survival rate below 10%, most patients present with advanced or unresectable disease (Bengtsson et al. 2020; Yu et al. 2021). In contrast to many other solid tumors, PDAC lacks broadly effective targeted therapies; only rare genomic subsets benefit from precision medicines (Wei and Ren 2024). Consequently, treatment still relies heavily on cytotoxic agents such as oxaliplatin (OXA). However, the dense desmoplastic stroma and poor vascular perfusion severely restrict drug penetration and intratumoral accumulation (Neesse et al. 2015; Bulle and Lim 2020; Ramaswamy et al. 2022; Shah et al. 2024), while systemic distribution and dose-limiting toxicities further limit clinical benefit (Rehman et al. 2022; Shah et al. 2024). These delivery barriers have motivated the exploration of alternative drug carriers. Among emerging strategies, extracellular vesicles (EVs) have drawn intense interest because of their nanoscale size, intrinsic biocompatibility, and natural ability to traverse biological barriers (Kalluri and LeBleu 2020). Multiple studies highlight their therapeutic versatility in oncology (Yang et al. 2024). For example, Yu Zhou and colleagues reported that MSC-derived EVs co-loaded with GEMP and PTX has already demonstrated superior apoptosis induction in MiaPaca-2 cells compared with conventional formulations (Zhou et al. 2020). EVs have also been successfully applied to platinum-based therapeutics (Elsherbeny et al., 2023; Bagheri et al. 2024). Guannan Zhou and colleagues demonstrated that cisplatin can be incorporated into milk-derived EVs with an encapsulation efficiency of 18%, thereby enhancing drug delivery by promoting efficient cytosolic entry and bypassing endosomal trapping, ultimately strengthening antitumor activity in cisplatin-resistant ovarian cancer cells (Zhou et al. 2022). Collectively, these findings highlight EVs as a promising platform for improving the therapeutic performance of chemotherapeutic agents in PDAC.

Despite these advantages, expanding EV-based chemotherapy in PDAC critically depends on achieving active targeting. Unmodified EVs exhibit only modest innate tropism and cannot overcome PDAC's heterogeneous, stroma-rich microenvironment (Kung and Yu 2023; Sha et al. 2025), a setting in which the classical enhanced permeability and retention (EPR) effect is markedly attenuated due to limited vascularization and poor perfusion (Xie et al. 2020). As a result, a substantial fraction of drug-loaded vesicles is inevitably sequestered in the liver and spleen, reflecting clearance by the mononuclear phagocyte system (Zhang et al. 2023). To address this, EV surfaces can be engineered to display antibody fragments, thereby enabling tumor specific uptake and significantly enhancing delivery precision (Tran et al. 2020; Choi et al., 2022; He et al. 2022). As exemplified by the work of Jun Jiang and colleagues, where anti-EGFR scFv-decorated EVs maintained roughly double the fluorescence intensity of control EVs in vivo and improved therapeutic efficacy (Jiang et al. 2024). Identifying an appropriate receptor becomes therefore essential for translating antibody-guided EV systems into PDAC. In this context, Ephrin type-A receptor 2 (EphA2) emerges as a particularly promising target owing to its high expression in PDAC, its additional presence within stromal components, and its robust capacity for ligand-induced internalization (Boissier et al. 2013; Wilson et al. 2021; Duxbury et al. 2023). Together, these observations suggest that engineering EVs with anti-EphA2 antibodies to encapsulate oxaliplatin may offer a rational strategy to enhance intratumoral drug delivery and potentially overcome the longstanding limitations of conventional chemotherapy in PDAC.

Here, we report a HEK293T-derived extracellular vesicle (EV) platform engineered to display membrane-anchored anti-EphA2 Fab domains and encapsulate oxaliplatin (OXA) for PDAC therapy (Wang et al. 2022). The engineered EVs are expected to exhibit binding affinity toward EphA2-positive PDAC cells, efficient OXA loading, and enhanced cellular uptake. In vitro, they are anticipated to produce greater cytotoxicity than free OXA, while in vivo they are designed to induce pronounced tumor regression accompanied by increased apoptotic activity. This work will advance a tumor-targeted delivery strategy that directly addresses the persistent barriers of penetration, selectivity, and drug delivery that undermine the efficacy of current PDAC therapies.

## 2. Materials and methods

### 2.1. Cell lines and culture conditions

HEK293T cells were used for the production of engineered extracellular vesicles (EVs) and for lentiviral packaging. Human pancreatic ductal adenocarcinoma (PDAC) cell lines AsPC-1 and BxPC-3 were used for in vitro functional assays. All cell lines were obtained from the American Type Culture Collection (ATCC, Manassas, VA, USA). HEK293T cells were maintained in Dulbecco's modified Eagle's medium (DMEM; Gibco, USA), whereas AsPC-1 and BxPC-3 cells were cultured in RPMI-1640 medium (Gibco, USA). All media were supplemented with 10% fetal bovine serum (FBS; Gibco, USA), 100 U/mL penicillin, and 100 µg/mL streptomycin (T&H Co., Ltd., Taipei, Taiwan). Cells were incubated at 37 °C in a humidified atmosphere containing 5% CO₂, passaged every 2–3 days to maintain exponential growth, and used within 20 passages after thawing.

### 2.2. Plasmid construction and transfection

The DNA sequence encoding the mouse anti-human EphA2 Fab fragment (clone 3F7) was codon-optimized for human expression and synthesized into the pUC57 vector by full-length gene synthesis (Omics Biotechnology, New Taipei City, Taiwan). The insert was subcloned into the lentiviral expression vector pLKO_AS3 using NheI and AscI restriction sites, and the construct was verified by restriction enzyme digestion.

Lentiviral particles were produced in HEK293T cells using a three-plasmid system consisting of the transfer vector pLKO_AS3-membrane-bound anti-EphA2 Fab, the packaging plasmid pCMV-delta8.91, and the envelope plasmid pMD.G. Transfections were performed with Lipofectamine™ 3000 (Thermo Fisher Scientific, USA) according to the manufacturer's protocol. Viral supernatants were collected 48–96 h post-transfection, clarified, concentrated with 100 kDa MWCO centrifugal filters (Merck Millipore, USA), and stored at −80 °C.

For stable cell line generation, HEK293T cells were transduced with concentrated lentiviral particles in the presence of polybrene (8 µg/mL) and selected with puromycin (3 µg/mL; InvivoGen, USA) for 10–14 days. Surviving clones expressing the membrane anti-EphA2 Fab fragment (M-anti-EphA2 Fab HEK293T) were expanded for EVs production.

### 2.3. Extracellular vesicles isolation and physicochemical characterization

Extracellular vesicles were produced from HEK293T and M-anti-EphA2 Fab HEK293T stable cell lines. Cells were seeded at the density of 1 × 10^6^ cells/mL, incubated overnight, and then treated with serum-free PBS or PBS containing 100 µg/mL oxaliplatin (OXA; TRC-O845075, Toronto Research Chemicals, LGC Standards, Toronto, Canada) (Zhang et al. 2022). To enhance vesicle release, cultures were exposed to UV irradiation for 2 hours and maintained for 24 hours before harvesting supernatants, as previously reported that UV exposure increases EVs secretion (Shen et al. 2020). EVs were purified by sequential differential centrifugation (600 g for 10 minutes, 2000 g for 10 minutes, and 10,000 g for 30 minutes), followed by ultracentrifugation at 100,000 g for 90 minutes at 4 °C (Beckman Coulter Optima XPN, Type 70.1 Ti rotor) (Yang et al. 2020). Pellets were resuspended in 200 µL PBS and quantified by protein concentration using a NanoDrop spectrophotometer (Thermo Fisher Scientific, USA). Drug-loaded EVs were prepared under the same conditions and quantified as described in Section 2.5.

The size distribution and particle concentration of extracellular vesicles were analyzed by nanoparticle tracking analysis (NTA) using a ZetaView® Nanoparticle Tracking Analyzer (Particle Metrix, Germany). Purified samples were diluted in PBS, and measurements were performed at 25.0 ± 0.1 °C, pH 7.0, scanning 11 positions per sample with two cycles. Data were processed with ZetaView Software version 8.05.16 SP3, and mean particle size and particle concentration were recorded.

### 2.4. Extracellular vesicles immunophenotyping and protein characterization

Surface antibody expression in M-anti-EphA2 Fab HEK293T cells was assessed by flow cytometry. Cells (1 × 10^6^) were resuspended with 0.05% BSA/PBS and stained with FITC-conjugated goat anti-mouse IgG F(ab')₂ antibody (Elabscience, Cat. No. E-AB-1088, 1:500) for 30 minutes at 4 °C in the dark. Cells were washed, filtered through a 40-µm mesh, and analyzed on a FACSLyric™ cytometer (BD Biosciences, USA). Data were processed with FlowJo software (v10.8.1), and histograms were compared with unstained controls.

For western blot analysis, whole-cell lysates were prepared using RIPA buffer supplemented with protease and phosphatase inhibitors, while purified EVs and EVs-EphA2 were directly lysed. Protein concentrations were determined with a NanoDrop spectrophotometer. Equal amounts of protein (100 µg) were denatured in reducing buffer at 95 °C for 10 minutes, separated by SDS-PAGE, and transferred onto PVDF membranes. The 3-color prestained protein marker (Antec Bio, PMB1; 10–180 kDa) was used as the molecular weight marker. Membranes were incubated overnight at 4 °C with primary antibodies against CD9 (Rabbit mAb, CST, Cat. No. 98327, 1:400) and CD81 (Rabbit mAb, CST, Cat. No. 56039, 1:400), followed by HRP-conjugated goat anti-rabbit IgG F(ab')₂ (Jackson ImmunoResearch, Cat. No. 111-036-003, 1:5000). EphA2 Fab expression on engineered EVs was detected with HRP-conjugated goat anti-mouse IgG (H+L) (Millipore, Cat. No. AP308P, 1:5000). Bands were visualized using the Immobilon Western Chemiluminescent HRP Substrate (Merck Millipore, Cat. No. WBKLS0500) and imaged with a MiniChemi™ 610 imaging system (Beijing Sage Creation Science Co., Ltd., China).

### 2.5. Binding and uptake assays

The antigen-binding capacity of engineered EVs was evaluated by enzyme-linked immunosorbent assay (ELISA). Recombinant human EphA2 protein (0.5 µg/mL in 0.05 M NaHCO₃, pH 9.6; MedChemExpress, Cat. No. HY-P70336) was coated onto 96-well high-binding plates (SpectraPlate™ 96HB) at 4 °C overnight. Wells were blocked with 5% BSA/PBST at 4 °C overnight and washed three times with PBS. EVs samples (EVs, EVs-EphA2, and PBS control; 50 µL/well) were added and incubated for 1 hour at room temperature with gentle shaking. After washing, HRP-conjugated goat anti-mouse IgG F(ab')₂ (1:1000) was added for 1 hour at room temperature. Wells were washed three times, and 150 µL ABTS/H₂O₂ substrate solution (3000:1) was added for 45 minutes in the dark. Absorbance at 405 nm was measured using a microplate reader to assess the specific binding to EphA2.

EVs uptake was assessed by fluorescence tracing. EVs (500 ng) were mixed with Rhodamine B (0.5 mg/mL) in 400 µL sucrose/PBS (600 mM) and electroporated using an ECM 2001 electroporation system (BTX Harvard Apparatus, USA) at 500 V, 200 ms, single pulse. Free dye was removed by ultracentrifugation at 100,000 g for 90 minutes at 4 °C, and pellets were resuspended in 200 µL PBS. Rhodamine B-labeled EVs were stored at −80 °C protected from light.

For uptake studies, AsPC-1 or BxPC-3 cells (2 × 10^5^ cells/mL) were seeded into black, clear-bottom 96-well plates (Greiner Bio-One, Cat. No. 655090) and incubated overnight at 37 °C with 5% CO₂. Cells were treated with EVs/Rho, EVs-EphA2/Rho, or PBS control for 90 minutes at 37 °C, then fixed with 4% paraformaldehyde and counterstained with DAPI. After three PBS washes, images were acquired using an ImageXpress® PICO automated imaging system (Molecular Devices, USA) with DAPI and TRITC filters at 20 ×  magnification under identical exposure conditions. Fluorescence intensity and distribution were analyzed to evaluate EVs uptake.

### 2.6. Drug loading quantification by LC–MS/MS

Drug-loaded EVs were prepared as described in Section 2.3. Prior to LC–MS/MS analysis, OXA-loaded EVs were lysed with 2% SDS to disrupt vesicle membranes and release encapsulated oxaliplatin. Oxaliplatin was reconstituted in PBS immediately before use, and the amount of OXA encapsulated in EVs was quantified by LC–MS/MS (Waters ACQUITY UPLC system coupled to a Finnigan TSQ Quantum Ultra triple quadrupole mass spectrometer, Thermo Electron, San Jose, CA) with data acquisition using Xcalibur software. Electrospray ionization (ESI) was operated in positive mode with a 10 µL injection volume. Separation was performed on an ACQUITY UPLC BEH C18 column (130 Å, 1.7 µm, 2.1 × 50 mm) with a guard column (2.1 × 5 mm) at 40 °C and a flow rate of 200 µL/min, using 0.1% acetic acid in ddH₂O (A) and 0.1% acetic acid in acetonitrile (B) as the mobile phase. Quantification was carried out in SRM mode, monitoring m/z 398→306 (retention time 1.71 minutes, collision energy 26 V, lens voltage 54). External calibration curves showed excellent linearity (R^2^ = 0.9985).

Results were first expressed as OXA concentration (ppb) in extracellular vesicles preparations and then normalized to extracellular vesicles protein content to obtain drug loading (DL, ng OXA per µg extracellular vesicles). Protein concentration was additionally determined using a NanoDrop spectrophotometer (Protein A280 mode), which yielded an average of 7.87 mg/mL (7870 µg/mL), consistent with vesicle counts obtained by NTA (~5.8 × 10^9^ vesicles/µg protein).

To estimate the number of drug molecules per vesicle, DL values were converted using NTA-derived vesicle counts according to the following equation:

$$\mathrm{Molecules}\;\mathrm{per}\;\mathrm{vesicle}=\frac{\mathrm{DL}\times 10^{9}÷{{\mathrm{MW}}_{OXA}\times N}_{A}}{\mathrm{Vesicles}}$$

where MW_OXA_ is the molecular weight of oxaliplatin (397.3 g/mol) and N_A_ is Avogadro's constant (6.022 × 10^23^ mol^−1^). In this calculation, DL (ng/µg) was first converted to moles per µg extracellular vesicles, multiplied by N_A_ to obtain the number of molecules per µg, and finally divided by the NTA-derived vesicle count to yield molecules per vesicle.

### 2.7. In vitro cytotoxicity

The cytotoxic effects of engineered EVs on pancreatic cancer cells were evaluated using the ATPlite 1step™ Luminescence Assay System (Revvity, Cat. No. 6016736). AsPC-1 or BxPC-3 cells were seeded at 5 × 10^3^ cells per well in 96-well plates and incubated for 24 hours at 37 °C with 5% CO₂ to allow cell attachment. Cells were then treated with four groups: EVs, EVs-EphA2, EVs/OXA, and EVs-EphA2/OXA. Each treatment was applied at concentrations of 0.25, 0.5, and 0.75 mg/mL, with PBS as the negative control. Following a 90 minutes incubation at 37 °C, the EVs preparations were removed, fresh medium was added, and cells were further cultured for 48 hours. At the end of incubation, 100 µL ATPlite working solution was added per well and incubated for 10 minutes at room temperature in the dark. Luminescence was measured at 560 nm using a microplate reader, and cell viability of PBS-treated controls was defined as 100%.

### 2.8. Animal studies

Animal experiments for this study were conducted primarily between January 2025 and May 2025, in accordance with institutional guidelines and approved by the Institutional Animal Care and Use Committee (IACUC; Approval No. 112167). Six-week-old BALB/c nude mice were purchased from the National Laboratory Animal Center (NLAC, Taipei, Taiwan). A bilateral xenograft model was established by subcutaneous injection of AsPC-1 cells (1 × 10^7^, right flank) and BxPC-3 cells (1 × 10^7^, left flank) into the hind limbs. Tumor volumes were measured every three days using a caliper and calculated as length × (width)^2^ × 0.5 (mm^3^). When tumors reached approximately 50 mm^3^, mice were randomized into treatment groups and subjected to therapeutic or imaging studies. The sample size (*n* = 6 per group) was determined based on previous colorectal cancer and pancreatic cancer xenograft studies in our laboratory, as well as published EV-based drug delivery experiments, which consistently showed that group sizes of 5–8 animals are sufficient to detect biologically meaningful differences in tumor growth while minimizing animal use. No formal a priori power calculation was performed. For therapeutic evaluation, mice were divided into six groups (*n* = 6 per group): PBS control, free OXA, EVs, EVs/OXA, EVs-EphA2, and EVs-EphA2/OXA. EV formulations were administered intraperitoneally at 400 µg per mouse (approximately 20 mg/kg), while free OXA was administered at 4 mg/kg. All treatments were performed every two days for a total of three doses, and tumor volume and body weight were recorded every three days until day 27. No other confounders were controlled.

For in vivo imaging, EVs were fluorescently labeled with Rhodamine B as described in Section 2.5. Tumor-bearing mice were injected via the tail vein with either EVs/Rho or EVs-EphA2/Rho (5 mg/mL). Fluorescence imaging was performed using the IVIS Spectrum system (PerkinElmer, USA) from 0 to 270 minutes post-injection. Imaging parameters were set as follows: excitation filter 535 nm, emission filter 600 nm, exposure time 0.5 s, binning medium, f/stop = 8, and lamp level low. Mice were maintained under isoflurane anesthesia (Attane®, Panion & BF Biotech Inc., Taiwan) during imaging in a dark chamber. Images were analyzed using Living Image software (PerkinElmer), with tumors defined as regions of interest (ROI). Fluorescence signals were quantified as radiant efficiency ([p/s/cm^2^/sr]/[µW/cm^2^]) and presented as heat maps to evaluate the biodistribution and tumor accumulation of EVs.

### 2.9. Anesthesia and Euthanasia

Suckling mice were anesthetized with isoflurane prior to intracerebral injection. At the experimental endpoint, animals were euthanized by gradual-fill CO₂ inhalation, performed in accordance with the AVMA Guidelines for the Euthanasia of Animals and the institutional animal care standards of Kaohsiung Medical University.

### 2.10. Tumor tissue analysis

At the end of the study, subcutaneous tumor masses were excised, rinsed with PBS, and weighed to determine tumor burden. Tumor tissues were fixed in 10% neutral-buffered formalin (BioTnA, Cat. No. TABS06-1000, Kaohsiung, Taiwan), embedded in paraffin, and sectioned into 4-µm slices.

Apoptosis was assessed by TUNEL assay using the TUNEL Apoptosis Assay Kit (DAB; BioTnA, Cat. No. TAAP01D, Kaohsiung, Taiwan). Sections were deparaffinized, treated with hydrogen peroxide for 30 minutes to quench endogenous peroxidase, and digested with proteinase K for 5 minutes. Slides were incubated with background reducing buffer for 60 minutes at room temperature, TdT reaction buffer for 10 minutes, and TdT working buffer for 1 hour at 37 °C. Incorporated digoxigenin-labeled nucleotides were detected using mouse anti-dig antibody (1 hour, 37 °C) followed by HRP-conjugated anti-dig polymer (30 minutes, room temperature). Signals were developed with 20 × -diluted DAB for 1 minute and counterstained with hematoxylin. TUNEL-positive nuclei were quantified in at least three random high-power fields (HPFs) per tumor.

DNA damage was evaluated by immunohistochemistry (IHC) staining for γH2AX (Bioworld, Cat. No. BS9844M, 1:200). Following deparaffinization, antigen retrieval was performed in Tris-EDTA buffer (pH 9.0) at 95–100 °C for 12 minutes. Endogenous peroxidase was blocked with hydrogen peroxide for 10 minutes, and slides were then blocked with 5% BSA for 30 minutes. Sections were incubated with anti-γH2AX antibody for 2 hours at room temperature, followed by HRP-conjugated secondary antibody for 30 minutes. Signals were developed with DAB substrate for 20 minutes and counterstained with hematoxylin. γH2AX-positive nuclei were quantified in at least three random HPFs per tumor using ImageJ software (NIH, USA).

### 2.11. Statistical analysis

All experiments were performed in at least three independent replicates. All quantitative data are shown as mean ± SEM unless otherwise specified. Statistical analyzes were conducted using GraphPad Prism version 10.0 (GraphPad Software, San Diego, CA, USA). Differences between two groups were evaluated by unpaired two-tailed Student's t-test, while multiple group comparisons were analyzed by one-way analysis of variance (ANOVA) followed by Tukey's post hoc test. A *p*-value < 0.05 was considered statistically significant. Statistical significance in figures is indicated as follows: *p < 0.05; **p < 0.01; ***p < 0.001; ****p < 0.0001.

## 3. Results

### 3.1. Schematic diagram of study design

**Figure 1.**
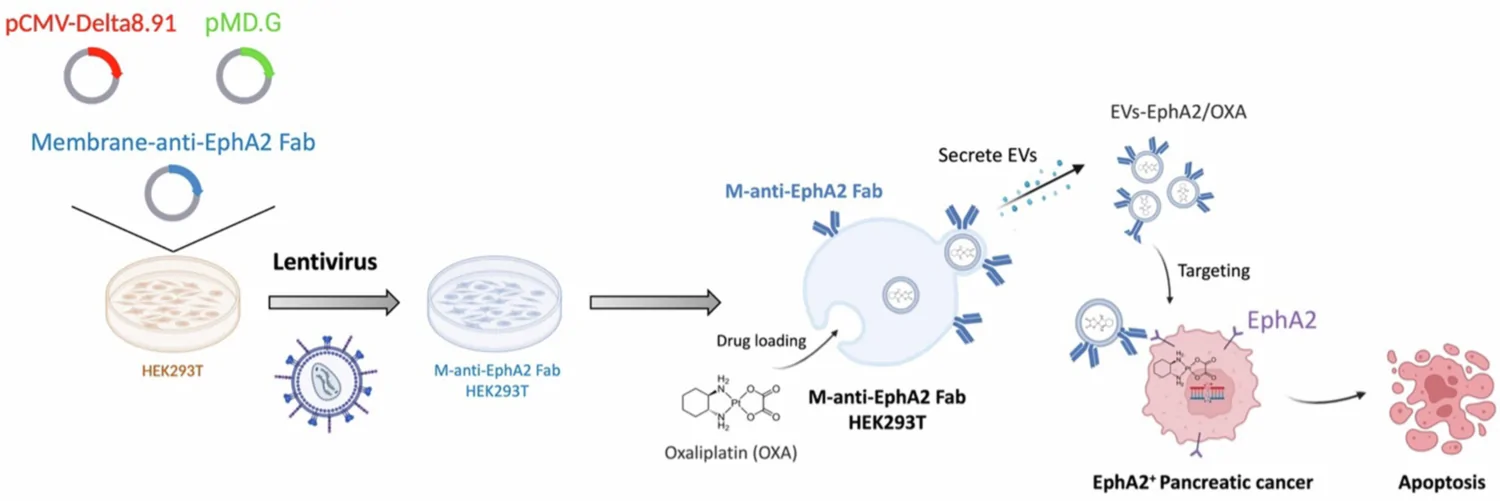
Schematic illustration of EVs-EphA2/OXA design. HEK293T cells were stably transduced with a lentiviral construct encoding a membrane-anchored anti-EphA2 Fab fragment to generate M-anti-EphA2 Fab HEK293T producer cells. These cells were subsequently exposed to oxaliplatin (OXA), enabling passive drug loading into secreted vesicles, which were then purified by ultracentrifugation. The resulting EVs-EphA2/OXA specifically bound to and induced cytotoxicity in EphA2⁺ PDAC cells both in vitro and in vivo.

We established a modular platform to generate EphA2-targeted, oxaliplatin-loaded extracellular vesicles (EVs-EphA2/OXA) for PDAC therapy (Figure 1). As in previous engineered extracellular vesicles strategies, proteins of interest were first expressed on the plasma membrane of donor cells and subsequently incorporated into secreted EVs (Ivanova et al. 2023; Jiang et al. 2024). In this study, HEK293T cells were stably transduced with a lentiviral construct encoding a membrane-anchored anti-human EphA2 Fab fragment to generate engineered EVs (EVs-EphA2). OXA was loaded through an endogenous loading strategy, in which producer cells were exposed to OXA-containing PBS under conditions, followed by EVs purification via ultracentrifugation (Yang et al. 2020). The resulting EVs-EphA2/OXA use the EphA2 antibody to guide vesicle entry into PDAC cells, which overexpress EphA2, delivering oxaliplatin directly into the tumors and inducing strong apoptotic responses, thereby providing a practical strategy for targeted pancreatic cancer therapy.

### 3.2. Generation and characterization of anti-EphA2 Fab-engineered extracellular vesicles encapsulating OXA

**Figure 2.**
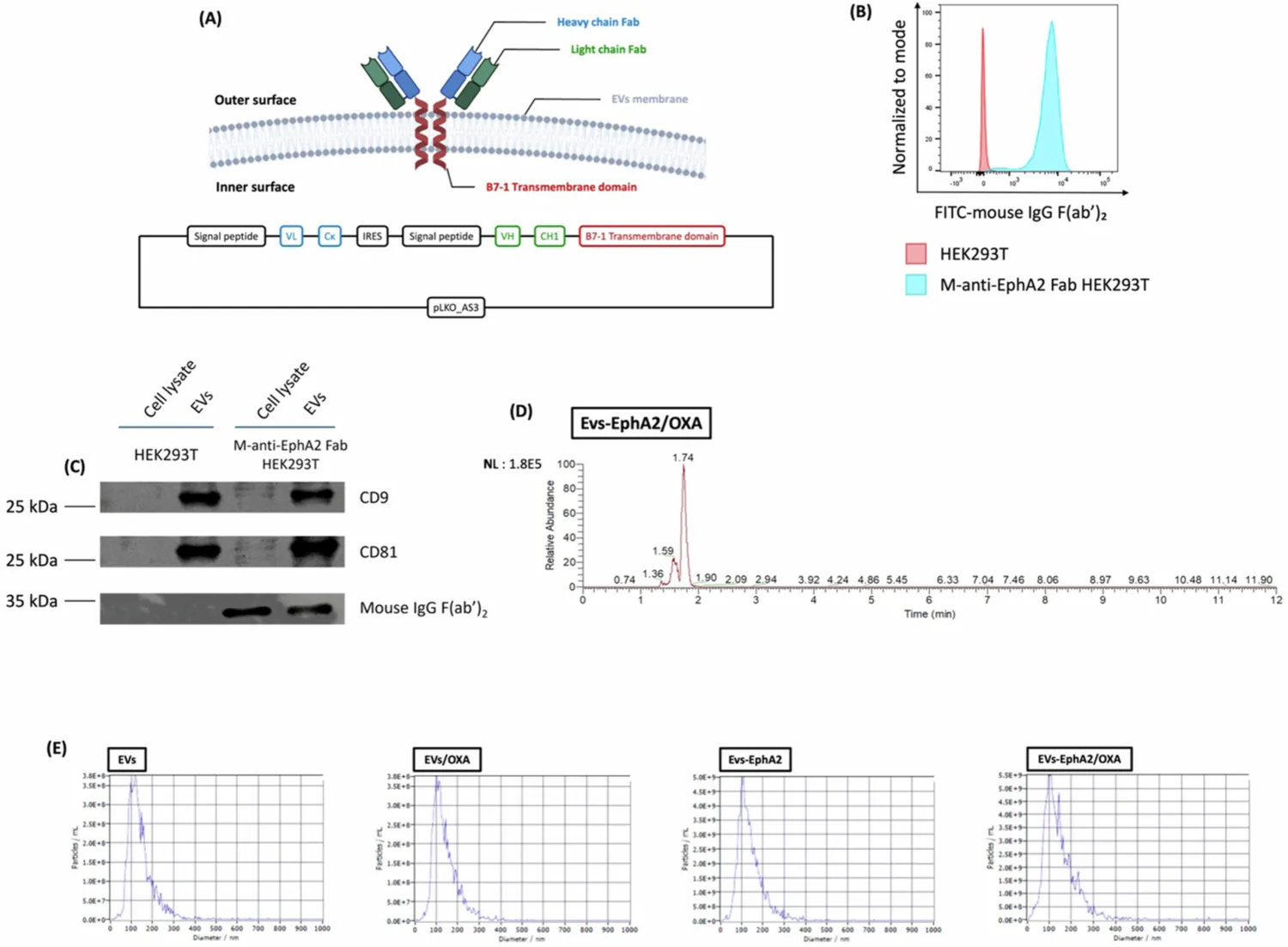
Generation and characterization of engineered extracellular vesicles (EVs-EphA2). (A) Schematic representation of the lentiviral construct encoding a membrane-anchored anti-EphA2 Fab fragment fused to the B7-1 transmembrane/cytoplasmic domain, ensuring stable surface display on producer cells and derived EVs. (B) Flow cytometry analysis confirmed robust surface expression of anti-EphA2 Fab in stably transduced HEK293T cells compared with parental controls. (C) Western blot analysis demonstrated the presence of CD9 and CD81 in all EV preparations, with engineered IgG fragments detected only in EVs-EphA2. (D) LC–MS/MS chromatogram demonstrating a distinct OXA peak at 1.74 minutes in EVs-EphA2/OXA, consistent with the retention time of authentic OXA standards, confirming successful drug encapsulation. (E) Nanoparticle tracking analysis showed that EVs, EVs/OXA, EVs-EphA2, and EVs-EphA2/OXA exhibited unimodal size distributions within the expected EVs size range, with mean diameters of 115.6, 105.1, 118.3, and 112.7 nm, respectively.

To generate EVs-EphA2, we designed a lentiviral construct encoding a membrane-anchored Fab fragment. The cassette was based on the 3F7 mouse anti-human EphA2 monoclonal antibody and comprised a signal peptide, light chain variable region (VL), constant *κ* domain (CK), an internal ribosome entry site (IRES), a second signal peptide, heavy chain variable region (VH), constant heavy chain domain 1 (CH1), and the transmembrane/cytoplasmic domain of human B7-1 (Figure 2A). This design enabled proper Fab assembly, detection, and stable anchoring to the plasma membrane, ensuring subsequent incorporation into secreted EVs (Lin et al. 2017). Stable expression of the membrane-bound Fab construct in HEK293T cells was confirmed by flow cytometry (Figure 2B). Cells transduced with the anti-EphA2 Fab lentiviral vector exhibited a marked rightward shift in fluorescence intensity, whereas parental HEK293T cells showed only background levels, confirming robust surface expression of the engineered Fab fragments.

After establishing stable producer cells, the secreted vesicles were isolated and characterized to verify successful engineering and drug loading. Western blot analysis confirmed that both EVs and EVs-EphA2 expressed canonical vesicular markers CD9 (~24–27 kDa) and CD81 (~26 kDa), validating particle isolation, whereas only EVs-EphA2 exhibited a distinct band corresponding to the engineered IgG fragment (~26–28 kDa) (Figure 2C). LC–MS/MS further verified oxaliplatin encapsulation in EVs-EphA2/OXA (Figure 2D), revealing a characteristic peak at 1.74 minutes co-eluting with authentic standards (Koellensperger and Hann 2010). Quantitative analysis indicated a drug loading of 0.391 ng OXA per µg EV protein, corresponding to approximately 118 molecules per vesicle based on NTA-derived particle counts. Nanoparticle tracking analysis demonstrated that all four formulations (EVs, EVs/OXA, EVs-EphA2, and EVs-EphA2/OXA) exhibited unimodal size distributions within the expected vesicular range (30–150 nm; mean diameters 115.6, 105.1, 118.3, and 112.7 nm, respectively) without evidence of aggregation (Boriachek et al. 2018; Kim et al. 2024) (Figure 2E). Collectively, these findings indicate that neither Fab anchoring nor oxaliplatin loading affected vesicle morphology, size distribution, or yield, establishing EVs-EphA2/OXA as a structurally and biochemically stable delivery platform for subsequent functional evaluation.

### 3.3. EVs-EphA2 achieve antigen-specific binding and selective uptake

**Figure 3.**
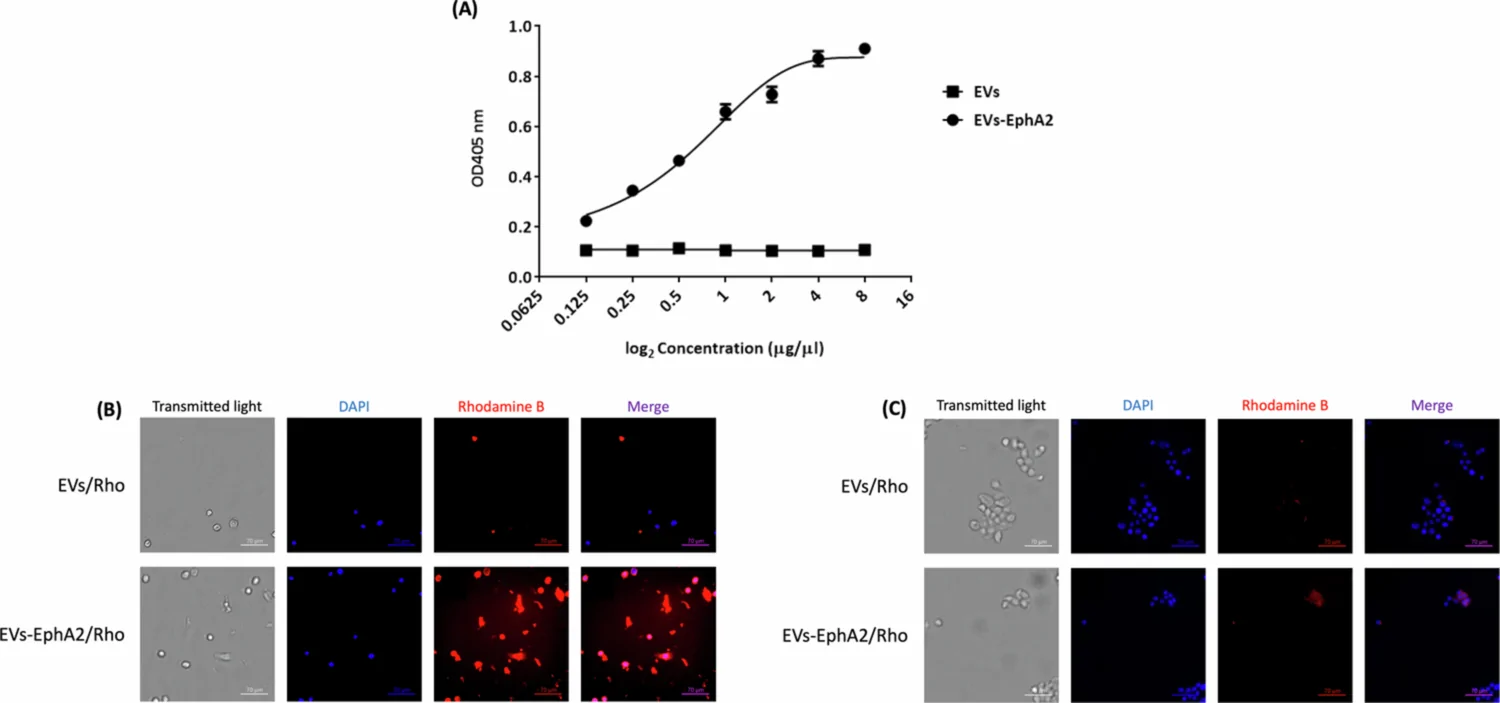
Antigen-specific binding and selective uptake of engineered extracellular vesicles (EVs-EphA2). (A) ELISA demonstrated dose-dependent binding of EVs-EphA2, but not control EVs, to recombinant EphA2. (B) Fluorescence microscopy showing uptake of Rhodamine B–labeled EVs-EphA2 into AsPC-1 cells, whereas control EVs exhibited minimal uptake. (C) Uptake of Rhodamine B–labeled EVs-EphA2 into BxPC-3 cells, with weaker fluorescence intensity compared to AsPC-1, consistent with lower EphA2 expression. Nuclei were counterstained with DAPI (blue). Scale bars, 70 µm.

To validate the functional display of anti-EphA2 Fab fragments on engineered EV surface, we first examined their antigen-binding capacity using ELISA. EVs-EphA2, but not unmodified EVs, bound recombinant human EphA2 in a dose-dependent manner, confirming that the engineered Fab fragments retained antigen recognition on the vesicle surface (Figure 3A). To further verify the binding specificity, fluorescence imaging revealed that Rhodamine B–labeled EVs-EphA2 were selectively internalized into EphA2⁺ PDAC cells, whereas control EVs and NC samples displayed only weak background signals. Specifically, in AsPC-1 cells (Figure 3B), EVs-EphA2 showed strong cytoplasmic red fluorescence, while in BxPC-3 cells (Figure 3C), uptake was relatively weaker, consistent with the lower EphA2 expression level in BxPC-3 (Supplementary figure 1). This positive correlation between receptor density and vesicle internalization efficiency further supports the antigen-specific interaction of the engineered particles. To further evaluate receptor-mediated EV uptake, flow cytometry analysis was performed using rhodamine B–labeled EVs. As shown in Supplementary Figure 2, treatment with EVs-EphA2/Rho resulted in a markedly higher Rhodamine B–positive cell population (52.9%) compared with EVs/Rho (21.5%). Importantly, this enhanced uptake was substantially reduced in the presence of a competing anti-EphA2 antibody (clone 3F7) (20.2%), consistent with EphA2-mediated internalization. Together, these data further support the enhanced cellular uptake of anti-EphA2–engineered EVs in AsPC-1 cells.

Collectively, these findings demonstrate that surface-displayed Fab fragments endow EVs with selective recognition and uptake toward EphA2-expressing PDAC cells.

### 3.4. Cytotoxic effects of OXA-loaded engineered EVs in EphA2⁺ PDAC cells

**Figure 4.**
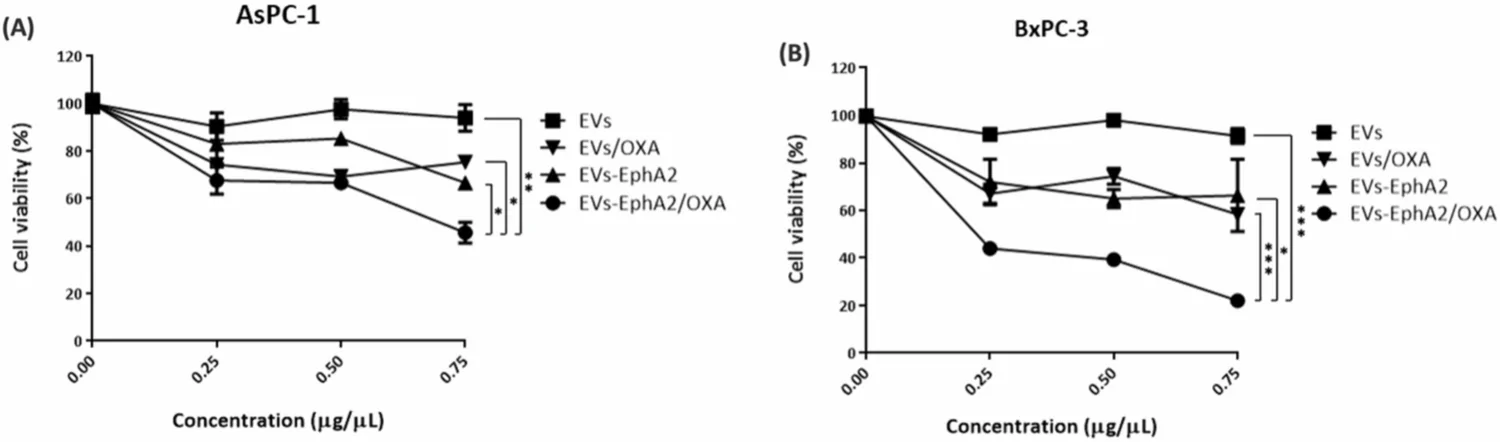
Cytotoxic effects of OXA-loaded engineered extracellular vesicles (EVs-EphA2/OXA). (A) Cell viability of AsPC-1 cells treated with EVs, EVs-EphA2, EVs/OXA, or EVs-EphA2/OXA at the indicated protein concentrations. (B) Cell viability of BxPC-3 cells treated under the same conditions. Unloaded vesicles (EVs, EVs-EphA2) had minimal effects on viability, whereas OXA-loaded vesicles induced a concentration-dependent cytotoxic effect. EVs-EphA2/OXA showed the strongest cytotoxicity in both cell lines. The indicated concentrations represent EV protein concentrations.

We next evaluated the cytotoxic activity of OXA-loaded EVs in EphA2⁺ PDAC cell lines. AsPC-1 and BxPC-3 cells were treated with EVs, EVs-EphA2, EVs/OXA, or EVs-EphA2/OXA. As shown in Figure 4A–B, EVs exhibited negligible effects on cell viability, confirming the high safety and biocompatibility of the vesicular carrier. In contrast, EVs-EphA2/OXA caused a dose-dependent decrease in cell viability, demonstrating effective intracellular drug delivery. Notably, EVs-EphA2/OXA produced markedly stronger cytotoxicity than EVs/OXA and OXA alone in both PDAC cell lines (Supplementary figure 3), consistent with enhanced uptake and drug retention mediated by EphA2-specific targeting. These findings indicate that antibody functionalization significantly potentiates the therapeutic efficacy of oxaliplatin while maintaining the inherent safety of the EV-based delivery platform.

### 3.5. In vivo antitumor efficacy of EVs-EphA2/OXA in PDAC xenograft models

To evaluate the therapeutic efficacy of engineered extracellular vesicles, AsPC-1 and BxPC-3 pancreatic cancer cells were subcutaneously xenografted into BALB/c nude mice and treated once tumors reached approximately 50 mm^3^. Six groups (*N* = 6 per group) were compared: PBS, free oxaliplatin (OXA, 4 mg/kg), OXA-loaded extracellular vesicles (EVs/OXA and EVs-EphA2/OXA), and unloaded extracellular vesicles (EVs and EVs-EphA2). EV formulations were administered intraperitoneally at 400 µg per mouse (≈20 mg/kg) every other day for three doses, with tumor volumes monitored until day 27.

**Figure 5.**
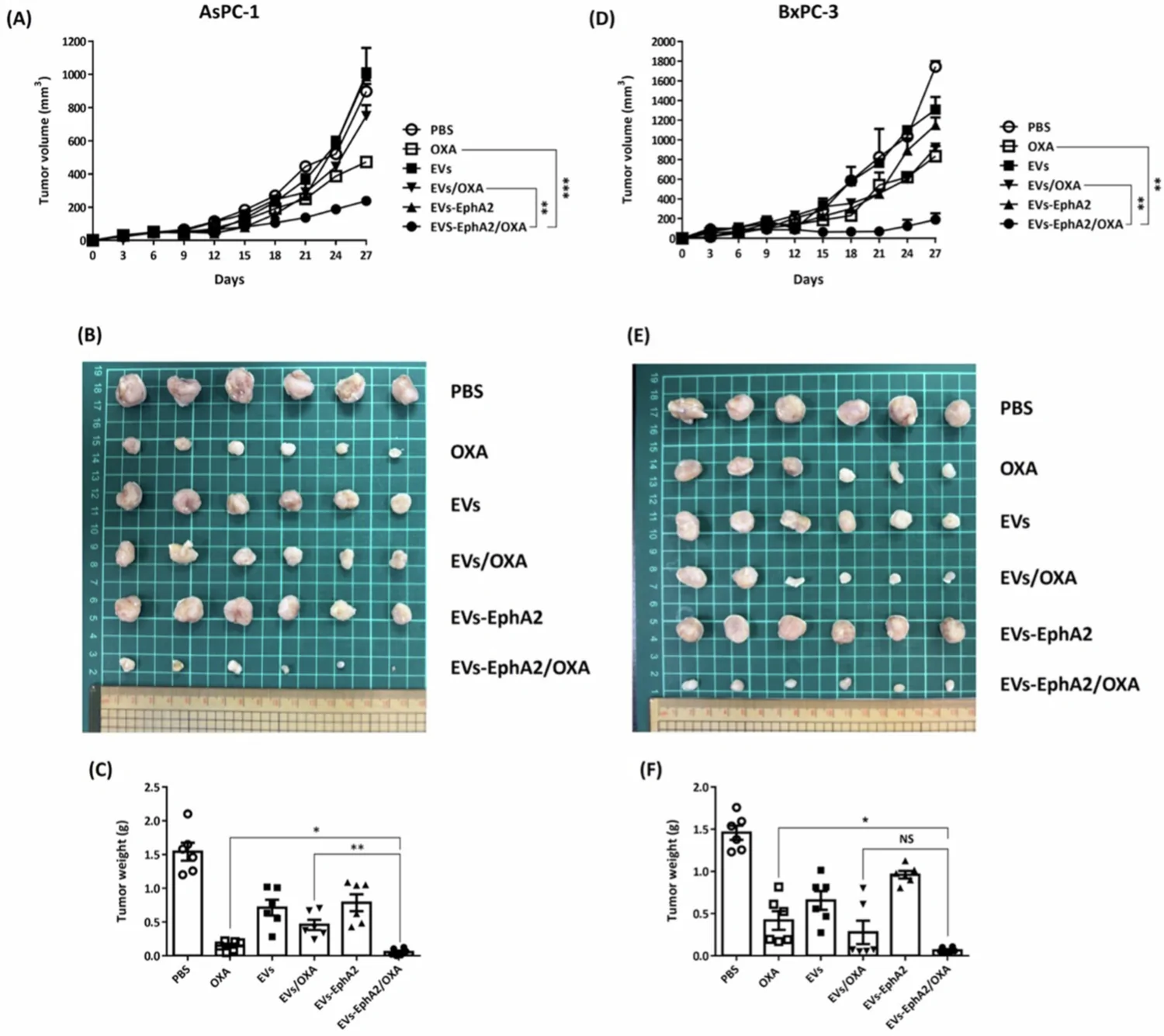
In vivo antitumor efficacy of EVs-EphA2/OXA in PDAC xenografts. (A) Tumor growth curves of AsPC-1 xenografts treated with PBS, free OXA, EVs, EVs/OXA, EVs-EphA2, or EVs-EphA2/OXA (*N* = 6 per group). (B) Representative images of excised AsPC-1 tumors at day 27. (C) Final tumor weights of AsPC-1 xenografts. (D) Tumor growth curves of BxPC-3 xenografts under the same treatments. (E) Representative images of excised BxPC-3 tumors. (F) Final tumor weights of BxPC-3 xenografts.

EVs-EphA2/OXA treatment produced the most pronounced tumor suppression in both xenograft models, significantly reducing tumor growth curves compared with all controls (Figure 5A and D). Representative tumor images confirmed markedly smaller tumor sizes in the EVs-EphA2/OXA groups (Figure 5B and E). Final tumor weights were reduced to 0.056 ± 0.05 g in AsPC-1 xenografts (Figure 5C) and 0.066 ± 0.03 g in BxPC-3 xenografts (Figure 5F), compared with 0.151 ± 0.07 g and 0.419 ± 0.27 g for free OXA, respectively. Tumor growth inhibition (TGI), calculated relative to PBS controls, demonstrated that EVs-EphA2/OXA achieved a 96.3% reduction in AsPC-1 and 95.3% in BxPC-3, substantially outperforming both free OXA (62.9% and 84.2%) and untargeted EVs/OXA (87.8% and 76.2%). Together, these results demonstrate that EVs-EphA2/OXA achieves markedly superior antitumor efficacy compared with both free OXA and non-targeted EV formulations, underscoring the advantage of EphA2-mediated targeting for enhancing chemotherapeutic delivery and tumor suppression in PDAC models.

These findings parallel the enhanced cytotoxicity observed in vitro (Figure 4), where EVs-EphA2/OXA more effectively eliminated EphA2⁺ PDAC cells compared with non-targeted counterparts. Consistent with these observations, in vivo fluorescence imaging (IVIS) of Rhodamine B–labeled EVs demonstrated preferential accumulation of anti-EphA2–engineered vesicles in tumor regions compared with unmodified EVs (Supplementary Figure 4). Moreover, mice treated with EVs-EphA2/OXA maintained significantly higher body weights than those treated with free OXA during the treatment period, suggesting reduced systemic toxicity of the engineered EV formulation (Supplementary Figure 5).

Together, these data demonstrate that EphA2 functionalization promotes tumor localization of engineered EVs, providing a mechanistic basis for their improved intratumoral delivery and superior therapeutic efficacy.

### 3.6. EVs-EphA2/OXA enhances intratumoral DNA damage and apoptosis in PDAC xenografts

**Figure 6.**
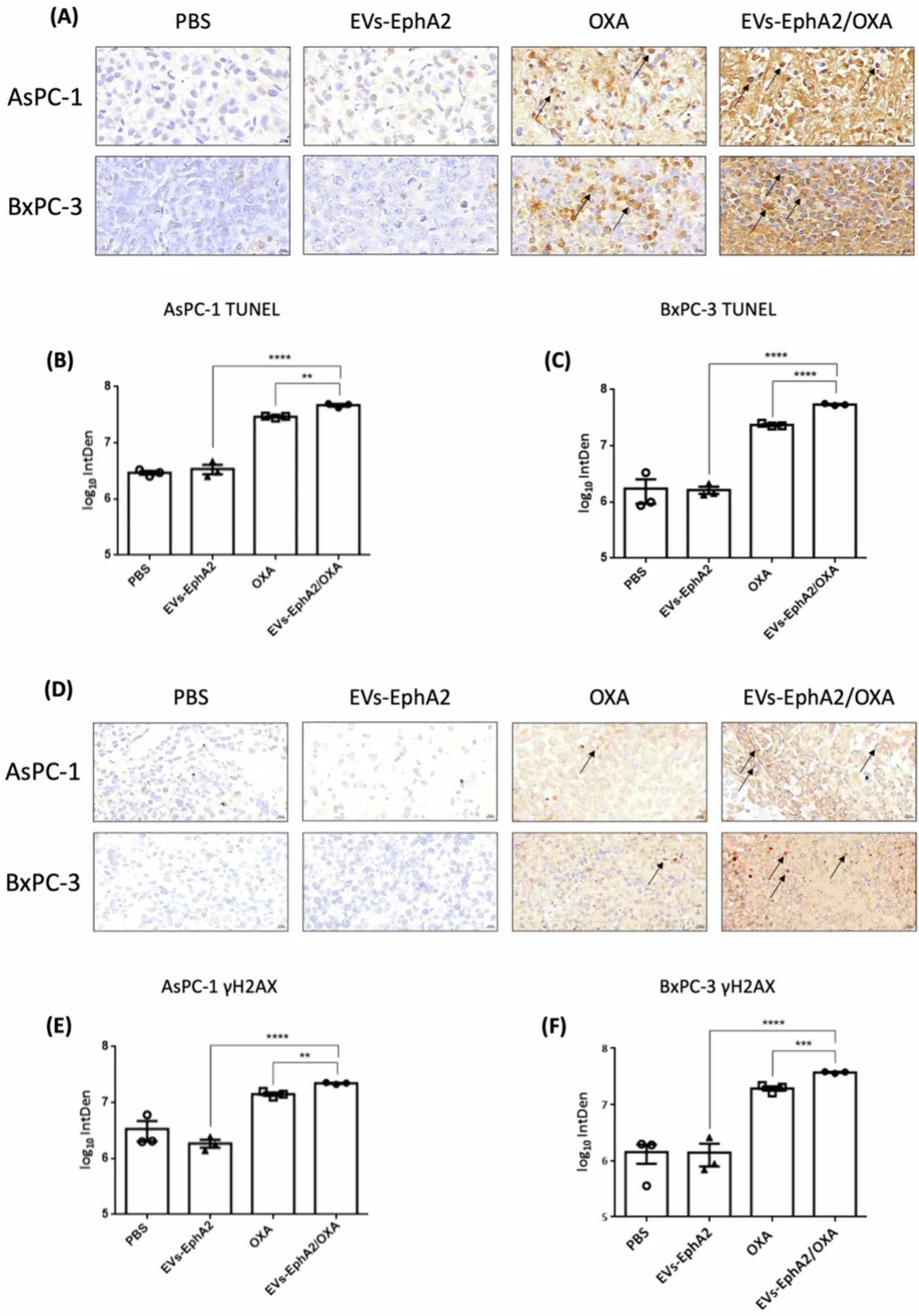
EVs-EphA2/OXA enhances intratumoral DNA damage and apoptosis in PDAC xenografts. (A) Representative immunohistochemical images of γH2AX staining in AsPC-1 and BxPC-3 tumor sections after treatment with PBS, EVs-EphA2, free oxaliplatin (OXA), or EVs-EphA2/OXA. Brown nuclear foci (arrows) indicate γH2AX-positive cells. Scale bars, 20 µm. (B, C) Quantification of γH2AX intensity (log₁₀ IntDen) in AsPC-1 and BxPC-3 xenografts. Both OXA and EVs-EphA2/OXA groups showed significantly stronger γH2AX signals than PBS and EVs-EphA2 controls. (D) Representative TUNEL staining of AsPC-1 and BxPC-3 tumor sections following the same treatments. Brown nuclei (arrows) denote apoptotic cells. Scale bars, 20 µm. (E, F) Quantification of TUNEL staining intensity (log₁₀ IntDen) in AsPC-1 and BxPC-3 xenografts. EVs-EphA2/OXA induced the highest apoptotic activity among all groups.

To evaluate whether EVs-EphA2/OXA enhanced oxaliplatin-induced cytotoxic injury in vivo, we first assessed apoptosis using TUNEL staining. Both PDAC xenograft models showed the highest apoptotic indices in tumors treated with EVs-EphA2/OXA, clearly exceeding the levels observed in free OXA, EVs-EphA2, and PBS groups (Figure 6A–C). These results indicate that targeted OXA delivery through EphA2-directed EVs leads to more extensive apoptotic cell death within tumors.

We next examined whether this apoptotic response was associated with increased platinum-induced DNA damage (Bonner et al. 2008). γH2AX immunohistochemistry revealed strong nuclear staining in tumors treated with free OXA or EVs-EphA2/OXA, whereas minimal signal was observed in control groups (Figure 6D). In AsPC-1 xenografts, EVs-EphA2/OXA produced a greater γH2AX signal than free OXA (Figure 6E), whereas BxPC-3 tumors exhibited similarly high γH2AX induction in both treatment groups (Figure 6F).

Together, these findings demonstrate that EphA2-targeted EVs potentiate oxaliplatin-mediated apoptosis and DNA damage in vivo, supporting the enhanced antitumor efficacy observed across PDAC xenograft models.

## 4. Discussion

In this study, we establish a modular extracellular vesicle (EV)–based nanocarrier system that integrates antibody-mediated tumor recognition with chemotherapeutic delivery. By engineering HEK293T cells to stably express a membrane-anchored anti-EphA2 Fab fragment, we generate EVs-EphA2 that retain antigen-binding capability and demonstrate selective uptake by EphA2⁺ PDAC cells. Oxaliplatin (OXA) is incorporated through an endogenous loading approach, yielding EVs-EphA2/OXA nanocarriers with quantifiable drug payloads and robust tumor-targeted cytotoxicity. Functional analyzes show that EVs-EphA2/OXA enhance in-vitro cytotoxic activity by more than 100-fold compared with free OXA and produce pronounced tumor suppression in PDAC xenograft models. Collectively, these findings highlight EVs-EphA2/OXA as a promising precision delivery platform for EphA2⁺ pancreatic cancer.

EVs are highly suitable carriers for diverse anticancer agents and have been increasingly explored as delivery vehicles to enhance chemotherapeutic efficacy (Elsharkasy et al. 2020). Compared with synthetic nanocarriers such as liposomes or polymeric nanoparticles, EVs express CD47 as a ‘don't-eat-me’ signal, exhibit reduced clearance by the reticuloendothelial system, and display innate tumor tropism, allowing more efficient penetration through desmoplastic stroma(Shi et al. 2017; Nam et al. 2020). Beyond oxaliplatin, EVs have also been reported to encapsulate a broad spectrum of cytotoxic drugs—including gemcitabine (Li et al. 2020), 5-Fluorouracil (Shekh et al. 2023), and doxorubicin (Tian et al. 2014) —demonstrating their flexibility as a modular platform for improving the pharmacological performance of traditional chemotherapies. In our study, we further leveraged antibody functionalization to endow drug-loaded EVs with tumor-specific recognition. Although unmodified OXA-loaded EVs (EVs/OXA) exhibited measurable antitumor effects (Figure 5), EVs-EphA2/OXA produced a markedly greater reduction in tumor burden. This comparison underscores a key principle in PDAC drug delivery: passive EV tropism provides some benefit, but antibody-engineered EVs are essential to achieve meaningful and consistent therapeutic gains in the highly stromal, poorly perfused PDAC microenvironment.

Ephrin type-A receptor 2 (EphA2) is an attractive target for PDAC because it is expressed in over 90% of PDAC cases (Chang et al. 2023). In addition, prior studies have demonstrated that EphA2-targeting antibodies can directly attenuate tumor progression by promoting receptor internalization and degradation and by suppressing oncogenic EphA2 signaling pathways (Carles-Kinch et al. 2002; Xiao et al. 2020). In a phase I clinical trial, the humanized anti-EphA2 antibody DS-8895a was shown to be safe and well tolerated in patients with advanced solid tumors, further confirming the feasibility of EphA2-directed therapies in the clinical setting (Hasegawa et al. 2016; Shitara et al. 2019). Beyond PDAC, EphA2 is also consistently upregulated across multiple solid tumors based on TNMplot database analysis (Supplementary Figure 6), including colorectal, gastric, lung cancers, as well as glioblastoma (Wykosky et al. 2005; Brantley-Sieders 2012). This broad overexpression further highlights EphA2 as a versatile targeting receptor suitable for antibody-engineered EV delivery systems across multiple cancer types.

Recognizing this broader landscape, our platform is intentionally designed not to be constrained by EphA2 alone. Because the membrane surface of OXA-loaded EVs can be modularly equipped with alternative antibody fragments, the same vesicle backbone can be redirected toward distinct tumor types simply by exchanging the targeting ligand. This modularity is not conceptual but functionally validated: in our previous work, EGFR-targeted EVs loaded with oxaliplatin (M-C-293EVs/OXA) achieved markedly enhanced cytotoxicity and significant tumor regression in EGFR⁺ colorectal cancer models (Chien et al. 2024). These findings demonstrate that Fab-engineered EVs retain targeting specificity, maintain drug-loading capacity, and preserve antitumor activity even when the surface receptor and tumor context differ. Together, these data highlight a central strength of the system—the ability to systematically repurpose a single EV chassis across diverse receptor-defined malignancies—positioning this approach as a scalable and adaptable platform rather than a receptor-restricted therapeutic construct.

Taken together, these considerations underscore that although EVs-EphA2/OXA represents a compelling therapeutic concept with clear preclinical efficacy, its successful translation will depend on resolving key manufacturing and biosafety hurdles (Lener et al. 2015; Thery et al. 2018; Li et al. 2019). Further optimization of EV drug loading efficiency, quantitative evaluation of batch-to-batch variability, and refinement of EV production and large-scale purification processes will be important for future translational development. At the same time, several features of our platform highlight its therapeutic promise: HEK293T-derived EVs exhibited favorable safety profiles in our previous studies, showing no detectable toxicity toward multiple types of normal human cells or tumor cells (Figure 4) (Chien et al. 2024); EphA2-functionalized EVs demonstrated receptor-dependent tumor accumulation in vivo, supporting the ability of surface engineering to impart strong tumor selectivity; and because EphA2 is broadly expressed across multiple solid tumors, the EVs-EphA2/OXA strategy may extend beyond PDAC to additional EphA2⁺ malignancies. Collectively, these attributes position antibody-guided EV nanocarriers as a feasible and adaptable platform that, with further alignment to GMP-compliant production standards, may ultimately advance the precision and therapeutic performance of chemotherapy in refractory solid tumors.

## Supplementary Material

Supplementary Material Supplementary Materials.docx

## Data Availability

The data supporting the findings of this study are available from the corresponding author upon reasonable request.
