# Supplementary material for: Bioengineered extracellular vesicles integrating anti-EphA2 recognition and oxaliplatin delivery for pancreatic cancer therapy
Source: Drug Deliv. 2026 Sep 22;33(1):2728215. doi: 10.1080/10717544.2026.2728215 (PMC13600275; doi:10.1080/10717544.2026.2728215)
Supplement: Supplementary file 1 [file IDRD_A_2728215_SM9903.docx]

**“Supplementary Materials”**

**Bioengineered Extracellular Vesicles Integrating Anti-EphA2 Recognition and Oxaliplatin Delivery for Pancreatic Cancer Therapy**

Shi-Wei Chao^#^, Wan-Chi Tsai^#^, Yung-Han Chang, Shan-Jou Chen, Chia-Tse Li, Jo-Han Fan, Bo-Cheng Huang, Shih-Ting Hong, Hong-Ren Wang, Yu-Ting Huang, Ming-Yii Huang^*^, Chih-Hung Chuang^*^

**Supplementary Figure 1. Flow cytometric validation of EphA2 expression in PDAC cell lines**

Surface expression of EphA2 was evaluated in AsPC-1 and BxPC-3 pancreatic cancer cells. Cells were incubated with primary anti-human EphA2 antibody followed by FITC-conjugated secondary antibody (blue), while isotype-matched IgG with FITC-secondary served as the negative control (red). Both AsPC-1 and BxPC-3 cells expressed detectable EphA2, but AsPC-1 displayed a markedly higher fluorescence intensity, indicating stronger EphA2 surface expression. This differential expression supports the observed higher uptake of ExoEph in AsPC-1 cells and validates both cell lines as relevant PDAC models for targeted delivery.

**Supplementary Figure 2. Quantitative analysis of EVs uptake by flow cytometry**

Flow cytometry histograms showing the percentage of Rhodamine B⁺ AsPC-1 cells after incubation with rhodamine B–labeled EVs (EVs/Rho), anti-EphA2–engineered EVs (EVs-EphA2/Rho), or EVs-EphA2/Rho in the presence of a competing anti-EphA2 antibody (clone 3F7). EVs-EphA2/Rho treatment resulted in a markedly higher Rhodamine B⁺ cell population (52.9%) compared with EVs/Rho (21.5%), whereas competitive inhibition with 3F7 reduced the Rhodamine B⁺ population to 20.2%, consistent with EphA2-mediated uptake of engineered EVs.

**Supplementary Figure 3. Cytotoxicity of free oxaliplatin in PDAC cell lines**

AsPC-1 (A) and BxPC-3 (B) pancreatic cancer cells were treated with increasing concentrations of free oxaliplatin (0–240 µM) for 48 h, and cell viability was measured using the ATPlite assay. Both cell lines exhibited clear dose-dependent cytotoxicity, with IC₅₀ values of 94.1 µM for AsPC-1 and 51.1 µM for BxPC-3. These results establish the baseline response of each PDAC model to oxaliplatin and provide a reference for evaluating EV-mediated drug delivery.

**Supplementary Figure 4. In vivo biodistribution of rhodamine B–labeled EVs in AsPC-1 tumor-bearing mice**

Representative IVIS fluorescence images showing the time-dependent distribution of rhodamine B–labeled extracellular vesicles (EVs/Rho) and anti-EphA2–engineered EVs (EVs-EphA2/Rho) in nude mice bearing subcutaneous AsPC-1 tumors. Fluorescence signals were recorded at the indicated time points (0–270 min) following administration. Regions of interest (ROI) were defined at the tumor site to evaluate vesicle accumulation. Compared with unmodified EVs, EVs-EphA2/Rho exhibited enhanced fluorescence intensity in the tumor region over time, indicating preferential tumor localization mediated by EphA2 targeting.

**Supplementary Figure 5. Body weight changes during treatment**

Body weight of mice receiving PBS, free oxaliplatin (OXA), EVs, EVs/OXA, EVs-EphA2, or EVs-EphA2/OXA during the treatment period. Body weight was monitored at the indicated time points as an indicator of systemic toxicity. Mice treated with EVs-EphA2/OXA maintained significantly higher body weights compared with the free OXA group, suggesting reduced systemic toxicity of the targeted EV-based oxaliplatin delivery system. Data are presented as mean ± SEM (n = 6).

**Supplementary Figure 6. EPHA2 gene expression across cancer types based on TNMplot database analysis**

Boxplots depict EPHA2 mRNA expression in paired normal (left) and tumor (right) tissues across multiple cancer types. Sample numbers for each group (N for normal; T for tumor) are shown beneath the plot. Cancer types marked in red (*) indicate a statistically significant difference between normal and tumor tissues (Mann–Whitney p < 0.05) combined with an absolute expression level >10 in at least one condition (tumor or normal). Data obtained from the TNMplot platform.
